# Investigating the effect of channel pruning on functional near-infrared spectroscopy data collected from children aged 5 to 24 months

**DOI:** 10.1117/1.NPh.13.1.015011

**Published:** 2026-02-14

**Authors:** Samuel Beaton, Borja Blanco, Chiara Bulgarelli, Clare E. Elwell, Sarah Lloyd-Fox, Ebrima Mbye, Samantha McCann, Anna Blasi Ribera, Sophie E. Moore

**Affiliations:** a King’s College London, Department of Women & Children’s Health, London, United Kingdom; b University of Cambridge, Department of Psychology, Cambridge, United Kingdom; c Birkbeck, University of London, Centre for Brain and Cognitive Development, London, United Kingdom; d University College London, Department of Medical Physics and Biomedical Engineering, London, United Kingdom; e Birkbeck University of London, Department of Psychological Sciences, London, United Kingdom; f Medical Research Council Unit, The Gambia at the London School of Hygiene and Tropical Medicine, Fajara, The Gambia

**Keywords:** infant fNIRS, channel pruning, processing

## Abstract

**Significance:**

Infant functional near-infrared spectroscopy (fNIRS) data are particularly vulnerable to noise; participant behavior can result in motion artifacts, and reduced set-up times can cause poor optode coupling. Accurate channel pruning is therefore essential, but approaches vary and often use adult-derived thresholds, risking unnecessary data loss.

**Aim:**

We systematically compared pruning approaches and parameter choices to evaluate their effects on data quality and retention in infant fNIRS.

**Approach:**

Data from 5 to 24-month-old infants were collected across two cohorts, using two paradigms. Channel pruning was performed using the coefficient of variation (CV) and the quality testing of near-infrared scans (QT-NIRS) tool, varying key thresholds. Multilevel models assessed the effects of pruning method, parameter choice, age, motion, and testing site on signal-to-noise ratio (SNR) and channels retained.

**Results:**

QT-NIRS produced significantly higher SNR than CV pruning across nearly all age, task, and cohort combinations when matched for data retention. Higher QT-NIRS thresholds improved quality but reduced retention. Motion prevalence strongly reduced both SNR and retention; testing site and age had smaller but notable effects.

**Conclusions:**

QT-NIRS offers a better balance of data quality and retention than CV pruning. Lower QT-NIRS thresholds than adult defaults are recommended for infant data. These findings provide practical guidance for preprocessing pipelines in developmental fNIRS research.

## 1 Introduction

### 1.1 Scalp-optode Coupling

In a typical functional near-infrared spectroscopy (fNIRS) experiment, participants wear a headband or cap embedded with optodes to monitor brain activity by emitting and detecting near-infrared light at two separate wavelengths. When fitted securely, the cap ensures a motion-robust signal, enabling the study of a wide range of cognitive tasks and abilities with less stringent demands for stillness than other neuroimaging modalities.^1^ For this reason, fNIRS is widely used with developmental populations.^2^ However, signal processing is usually still required to remove artefacts arising from motion, poor scalp-optode coupling, and physiological signal confounds.^3^^–^^5^ Infant data are particularly susceptible to motion and poor scalp-optode coupling, as infants typically exhibit increased fussiness, limited compliance with instructions, and shorter attention spans, which increase the likelihood of participant motion, reduced capping time, and difficulties in handling the imaging headgear.^6^ In fact, poor coupling allows light from the source optode(s) to escape, or ambient light to flood detector optode(s).^7^ Affected channels can exhibit signal saturation (easily detectable by unrealistically high raw intensity values caused by excessive light reaching the detector) and greater variability, which impacts the estimation of the hemodynamic response^8^ the amplitude of which is already lower and more variable in infants than in older participants.^9^^,^^10^

### 1.2 Strategies to Mitigate Poor Optode Coupling

Fitting the fNIRS headgear securely aids scalp-optode coupling^7^ but is time-consuming and assumes stable coupling throughout recording, which is challenging when working with infant participants. To reduce the impact of poor coupling on data quality, post-hoc channel pruning–the exclusion of data from an entire channel–is therefore often required. An important pruning consideration is the trade-off between data retention and quality: removing poorly coupled channels improves overall signal quality and mitigates the impact of superfluous signals but reduces the number of remaining channels and participants available for analysis.^6^ This is particularly important in infant research, given the already high attrition rates due to low attention span and susceptibility to fussiness.^11^ Pruning method selection is important, and manually pruning channels is subjective and time-consuming,^8^ especially for high-density imaging arrays. Two methods are often used to prune channels: the coefficient of variation (CV) or the “quality testing of near-infrared spectroscopy” (QT-NIRS) tool.

CV pruning quantifies the relative signal variability. Channels are pruned if the CV for either wavelength falls below a particular threshold,^12^^,^^13^ if the CV difference between wavelengths exceeds a threshold,^14^ or both.^15^ Although this method is faster and less subjective than manual pruning, a parameter choice is still required, and some levels of variability–which are expected in the task-based fNIRS signal due to the evoked hemodynamic response^16^–may be interpreted as signal noise. In addition, CV pruning only examines the signal in the time domain, potentially overlooking important signal properties.

QT-NIRS utilizes objective signal measures from both the time and frequency domains,^7^^,^^16^ providing a more comprehensive consideration of signal quality. Channels are pruned via signal characteristic assessments in two domains using the scalp coupling index (SCI) and peak spectral power (PSP). The SCI is a time-domain approach, which assesses correlation between wavelengths within the cardiac frequency band after bandpass filtering the signal, with high values indicative of a strong cardiac component.^17^ This incorporates a time-domain signal characteristic but also risks retaining channels with high correlation due to motion-induced artefacts. To address this, QT-NIRS incorporates a second measure based on the frequency domain, PSP, which detects strong, recurrent oscillations in the signal. High PSP values in the cardiac frequency band likely correspond to cardiac pulsations, whereas components with inconsistent or varying frequencies usually result in lower PSP values. QT-NIRS utilizes strengths from manual pruning (physiological grounding, consideration of both time- and frequency domains) and CV pruning (objectivity, efficiency), making it a robust tool for assessing data quality at the channel level.

Recently, QT-NIRS has been increasingly adopted in infant fNIRS research^18^^,^^19^ yet independent comparisons with other pruning approaches are yet to be established, and empirical estimations of SCI and PSP parameters are available only for adult participants.^7^^,^^16^ This highlights the need to refine its implementation with infant data. In addition to behavior, both physiological and anatomical factors may affect channel pruning in infants: they have thinner scalp tissue and higher cardiac signal frequencies (∼1.3 to 3.2 Hz at rest, compared with ∼1 to 1.7 Hz in adults).^20^^,^^21^ The former may result in a weak superficial cardiac signal, whereas the latter may result in coarse representations of the infant cardiac signal by fNIRS instrumentation sampling rates optimized for adult participants.^22^ Further, signal quality can be detrimentally affected by skin and hair color, hair type, age, and even head size, with darker skin pigmentation and thicker hair corresponding to poorer signal quality when compared with other skin and hair types,^23^ illustrating the need for inclusion of less frequently sampled populations in fNIRS studies.

Against this backdrop, the objectives of this work are to

a. Compare QT-NIRS as a pruning method against pruning using CV, which is used frequently by fNIRS users–and provides a baseline for channel pruning via a previously employed method and parameters.
b. Investigate contextual and data-derived measures that affect data quality and channel retention (with a particular focus on QT-NIRS parameter choices, age, and motion incidence).
c. Provide guidance on channel pruning and QT-NIRS use for infant participants.

To achieve this, fNIRS data from the Brain Imaging for Global HealTh (BRIGHT) Project,^24^ a longitudinal study of infant development in Kiang West (The Gambia) and Cambridge (UK), were analyzed. The analyses in this work incorporate data from two different experimental paradigms, collected from both the Gambian and UK sites and pertaining to participants with physical and behavioral characteristics from both a commonly sampled and a more underrepresented population in fNIRS research.^23^ The longitudinal nature of the data (with five time points over the first two years of life) further enables the investigation of the effects of age across early childhood while accounting for variability in cohort and task.

Based on prior literature and exploratory findings, the following hypotheses were formulated:

1. QT-NIRS-based pruning will result in a greater balance of data quality and retention compared with CV due to its multidomain examination of signal characteristics.
2. Motion will be negatively associated with signal quality and retention because of (i) increased likelihood of latent, undetected artifacts in data where more motion is detected, (ii) displacement of optodes affecting signal quality after motion artifacts, or both.
3. Data quality and retention will be diminished in the Gambian cohort, given the increased probability of darker, coarser hair types interfering with optode-scalp coupling.

## 2 Methods

### 2.1 Data

#### 2.1.1 Participants

Participants were recruited into the BRIGHT project from early 2016 to February 2018, and fNIRS data were collected when infants were 1-, 5-, 8-, 12-, 18-, and 24 months (hereafter x mo for x months of age), plus a follow-up between 3 and 5 years of age.^25^^,^^26^ The 1mo fNIRS protocol was limited to auditory stimuli with sleeping participants,^27^ likely inducing infant motion with a different noise profile; at 3 to 5 years, a different fNIRS cap for data collection was used, and data at this age were not collected in the UK site. To enable matched dataset comparisons, data from the other five time points (5-, 8-, 12-, 18-, and 24mo) were therefore used in this work. Participants met the inclusion criteria if infants were born at 37 to 42 weeks’ gestation (both cohorts) and had a minimum birth weight of 2.5 kg (UK only).

After applying exclusion criteria, a total of 204 mother-infant dyads were included in the Gambian cohort; of these, 185 remained at the 24mo timepoint. Pregnant, Mandinka-speaking women were recruited during routine antenatal clinical assessments at MRCG@LSHTM Keneba Field Station by fieldworkers in the Gambian BRIGHT Project team. An information sheet and consent form written in English were provided to potential recruits and then explained fully in Mandinka by a study staff member. fNIRS data collection took place at MRC Unit The Gambia at the London School of Hygiene and Tropical Medicine (“MRCG@LSHTM”) Keneba Field Station. Ethical approval was granted by the joint Gambia Government/MRC Ethics Committee under the title: “Developing brain function for-age curves from birth using novel biomarkers of neurocognitive function,” SCC number 1451v2.

Sixty-one mother-infant dyads were enrolled in the UK from the Rosie Hospital, Cambridge University Hospitals NHS Foundation Trust. Information about BRIGHT was provided during antenatal appointments, with families expressing an interest contacted and recruited subsequently via email or phone call. Data collection primarily took place at Evelyn Perinatal Imaging Centre at Rosie Hospital, Addenbrooke’s Hospital, Cambridge, and to a lesser extent at the Centre for Brain and Cognitive Development in Cambridge.^24^^,^^28^ Ethical approval was given by the National Research Ethics Service Committee East of England (REC reference 13/EE/02000); informed written consent was obtained from all parents/carers prior to participation.

#### 2.1.2 fNIRS paradigms

##### Social/NonSocial paradigm

Full details of the social/nonsocial (SNS) task paradigm can be found in Refs. 15, 29. Briefly, the paradigm consisted of alternating visual social (silent), auditory social, and auditory nonsocial stimuli. Stimuli were repeated until the participant became bored or fussy or the end of the task was reached; inter-stimulus baselines varied between 9 and 12 s.

Visual social stimuli consisted of full color, life-sized videos of adults from the same population as the participant on a 24-inch screen ∼100  cm away. Throughout, adult actors in the video either moved their eyes or played “hand-games” for 9 to 12 s. Actors, their actions, and concurrent spoken auditory stimuli were varied to prevent anticipatory brain activity. Auditory stimuli were nonsynchronized to the video in terms of both duration (8 s) and content, with environmental sounds for nonsocial stimuli and nonvocal speech sounds for social stimuli. Sounds in each condition (social and nonsocial) were matched for duration and sound intensity.

##### Habituation and novelty detection (HaND) paradigm

The experimental paradigm included 25 trials, each consisting of a spoken 8 s sentence in the family’s first language (i.e., English or Mandinka) followed by 10 s of silence. The first trial was preceded by at least 10 s of silence, acting as a baseline.

The same recording, with a female voice, was used for trials 1 to 15; a different, male voice was used for trials 16 to 20; finally, the original, female recording was again used for trials 21 to 25. The stimulus sentence: “Hi baby! How are you? Are you having fun? Thank you for coming to see us today. We’re very happy to see you” was translated to Mandinka to maintain the same semantic meaning.

Technical details on the recording, processing, and playback of the auditory stimulus can be found in previous work.^14^^,^^26^

#### 2.1.3 Data collection

**Fig. 1:**
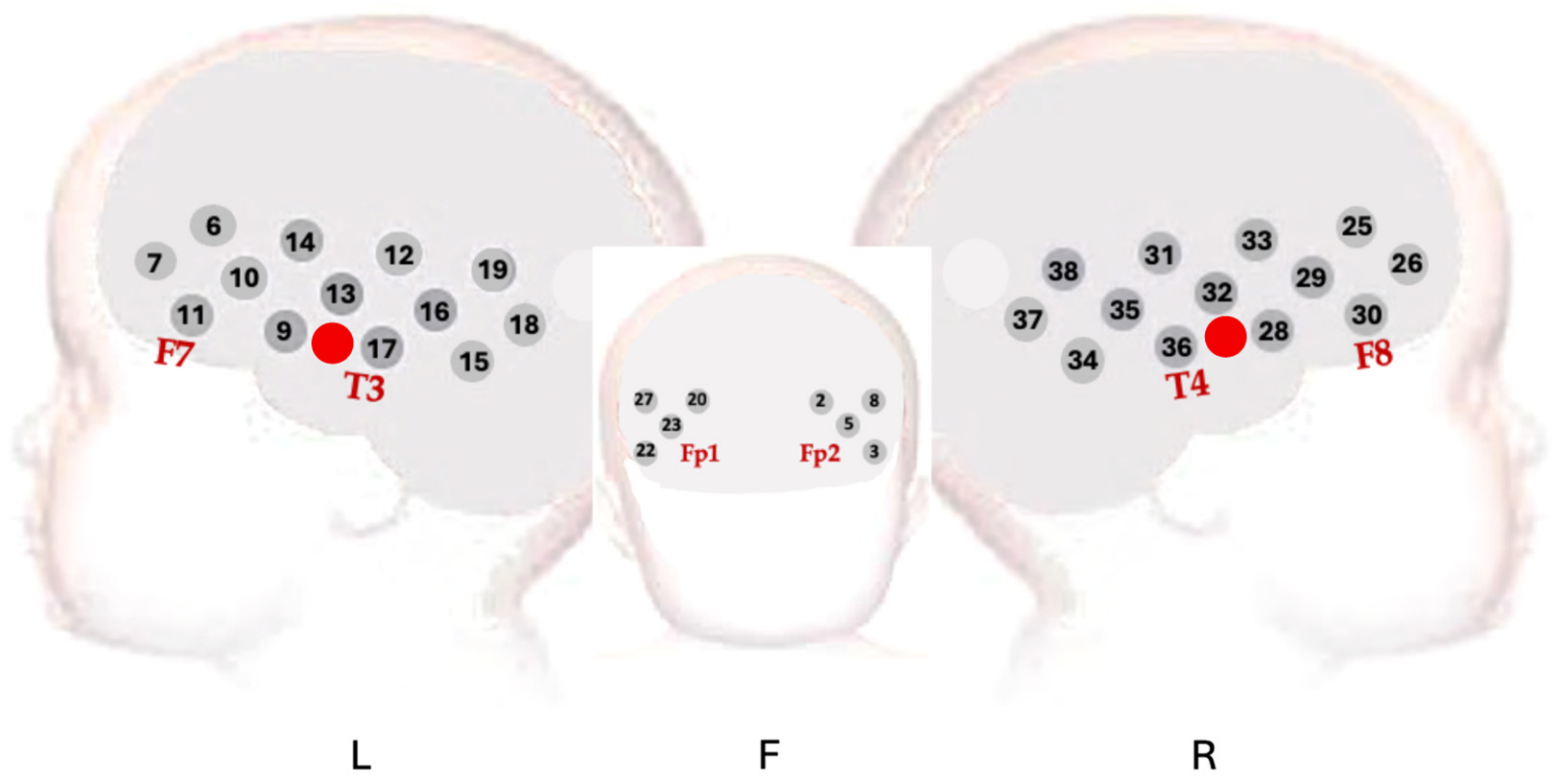
Array layout during data collection. Red dots indicate the position of optodes centered above the preauricular point during headgear fitting.

Custom-made headgear was fitted after head measurements (head circumference and ear-to-ear, both around the forehead and over the top of the head) had been taken to aid with the alignment of fNIRS headgear with the 10/20 system anatomical landmarks. Headgear consisted of custom-built stretchy silicone headbands to increase friction and prevent slippage, with attached probes into that optodes were clipped. Optodes were designed to accommodate glass optic fibers at right angles to allow them to sit flush on the scalp. The headband was fastened around the head to provide even pressure over the base of the probes.^30^^,^^31^ In the left hemisphere, headgear was placed such that source 4 in Fig. 1 was centered above the preauricular point, so that the channel it formed with the detector located directly behind it sat above T3 in the 10 to 20 system; the equivalent right hemisphere channel was above T4. The array angle was guided by the headband, which was placed on the head so that it touched the join between the ear and head and, frontally, lay over the infant’s brow line (through Fp1 and Fp2 in the 10 to 20 system).^32^

The headgear was designed to record responses bilaterally from auditory-associative brain regions, including the inferior frontal gyrus (IFG), middle and superior temporal regions, and the temporo-parietal junction.^14^^,^^33^^,^^34^ The fNIRS array comprised 17 channels in each hemisphere with a source-detector (S-D) distance of 2 cm, corresponding to a penetration depth of ∼1  cm from the skin surface and thus permitting measurement of the gyri and superficial sulci.^14^ fNIRS data were collected using this array design with the NTS optical topography system (Gowerlabs Ltd., UK) with a sampling frequency of 10 Hz and source wavelengths of 850 and 780 nm.

Infants sat on a carer’s lap during data acquisition. Carers were discouraged from interacting with the infant to attempt to minimize confounding stimuli; however, infants’ attention was engaged, if necessary, with (nonsocial and nonauditory) bubble-blowing and silent demonstration of soft toys, which also minimized infant head movement. The HaND task was part of a larger battery of fNIRS assessments with a total recording time of ∼21  min 30 s (6 min social task; 4 min functional connectivity data acquisition; 7 min 30 s HaND; and 4 min further functional connectivity). Where possible, paradigms were completed uninterrupted; sessions were paused and subsequently resumed in the event of infant discomfort.^35^

### 2.2 Channel Pruning

#### 2.2.1 Prepruning steps

First, channels were excluded from analyses if their minimum light intensity value was less than 3e-4, based on previous experience with the NTS system^14^; these were labeled “channels with signal extrema” (CSE). Analysis of the pruning methods was conducted on motion-free segments. To find motion-free data, motion artifacts were detected using hmrMotionArtifactByChannel function from Homer2^36^ with established infant fNIRS preprocessing parameters: tMotion = 1, tMask = 1, STDEVthresh = 15, and AMPthresh = 0.4.^37^ Data for each channel was split into 3 s windows per channel, as this aligned with QT-NIRS temporal segmentation and thus avoided additional processing complexities. Windows were excluded from pruning analyses if they contained artifacts at any point. If the data for a particular channel at one wavelength was excluded, data for both wavelengths were removed.

The two-channel pruning methods assessed in this work were implemented using custom-written scripts in MATLAB.^26^ Both pruning methods described use raw light intensity data as input.

#### 2.2.2 CV Pruning

CV pruning was conducted using an in-house script, and CV itself was calculated on motion-free data for each wavelength and channel using the equation:

$$CV=\frac{\sigma }{\left|\mu \right|},$$

where σ and μ are the standard deviation and mean of the light intensity signal in a channel, respectively.^38^ Channels were pruned if the difference between CV values for each wavelength exceeded 0.2 based on previous experience with the same data and system.^14^

#### 2.2.3 QT-NIRS pruning

QT-NIRS was implemented using the function qtnirs (available at https://github.com/lpollonini/qt-nirs at the time of writing) for precise control over the pruning and quick, repeated processing of the large volume of data.

More detail on QT-NIRS can be found in publications describing the methods,^7^^,^^17^ but an outline is provided here. First, bandpass filtering is conducted to retain only those frequencies in the cardiac band, ∼1.3 to 3.2 Hz in the case of infants.^20^ The cross-correlation of contemporaneous (zero-lag) wavelength signals in the cardiac frequency band (i.e., SCI) is then calculated:

$$\mathrm{SCI}={\overset{¯}{x}}_{\lambda 1}⊗{\overset{¯}{x}}_{\lambda 2}(0),$$

where ${\overset{¯}{x}}_{\lambda i}$ represents the light intensity signal for wavelengths i=1,2 in a motion-free signal. PSP is the maximum signal value in the frequency domain, representing the dominant oscillation in the bandpass-filtered signal and presumed to correspond to the cardiac frequency in well-coupled data. The recorded signal is divided into equal-length windows, and both SCI and PSP are calculated for each window. A window’s signal is considered of sufficient quality if both the calculated SCI and PSP exceed the user-defined thresholds, sci_threshold and psp_threshold, respectively.

The focus was on the alteration of sci_threshold and psp_threshold during analysis, since each provides a threshold for one of the key measures of optode coupling quality used to assess data quality with QT-NIRS, and adult reference values of sci_threshold = 0.8 and psp_threshold = 0.1 are available for these two parameters.^16^ Default parameters were used for window size (3 s) and quality threshold, or q_threshold (0.75), which prunes channels with less than 75% of windows meeting both SCI and PSP threshold values.

### 2.3 Statistical Analyses

Multilevel models (MLMs), a form of linear regression that estimates variance at multiple levels, were used for statistical analyses to effectively account for repeated measures, hierarchical data structures (including participant- and channel-level measures), and missing data.^39^ All models were fitted in R 4.4.1^40^ using the lme4 package.^41^ All models included random intercepts for each participant to account for individual variation. Final models used for analysis are described in Sec. 2.3.2.

#### 2.3.1 Measures, outcomes, and effects

Considering the anticipated data quality/inclusion trade-off, which is central to decision-making around preprocessing of fNIRS data,^6^ the performance of pruning methods and parameters was assessed using two metrics: (i) signal-to-noise ratio (SNR) and (ii) channel inclusion/exclusion percentage.

The effects of other factors, such as age, motion, and signal extrema, were included as predictors. Other measures were more particular to this dataset–such as task, cohort, and optode position. They were included as covariates to account for the variability they may cause.

The variables used are listed below, with letters contained in brackets indicating whether they were used as outcome variables (O), predictors (P), or covariates (C). Full rationale can be found in Supplementary Material 1.

##### Task-relevant channel signal-to-noise ratio (O)

Signal quality after pruning was measured on the included channels using the SNR:

$$\mathrm{SNR}=20\;\log_{10}\;\frac{\mu }{\sigma },$$

with μ and σ the mean and standard deviation of the signal, respectively.^5^ SNR was calculated in “task-relevant channels” (TRCs)—channels where a true hemodynamic signal was observed. Age-specific TRCs were determined using prior analyses: the SNS TRCs were taken from work by Benerradi and colleagues,^25^ whereas the HaND TRCs were taken from work by Blasi and colleagues.^26^ TRCs for each age and task were those that exhibited a hemodynamic response in both chromophores for either cohort, except the SNS task at 18mo: only one channel met this criterion, so channels were added for this age/task combination if they were in the set of TRCs for at least two other ages for the SNS task. This outcome was named the task-relevant channel signal-to-noise ratio (TRC SNR).

##### Channels retained (O)

The number of channels per participant included after pruning using the described method and parameter(s) was summed—this was labelled “Channels Retained” (CR).

##### SCI threshold (P)

sci_threshold values ranging from 0.05 to 0.9 were used, with increments of 0.05. The upper threshold of 0.9 sits between the recommended value for adult participants (0.8) and the theoretical maximum of SCI = 1. Initial exploratory analyses (see Supplementary Material 2) for each age/task/cohort combination indicated that even very low SCI values continued to alter signal quality, so the entire range of lower values was used.

##### PSP threshold (P)

**Fig. 2:**
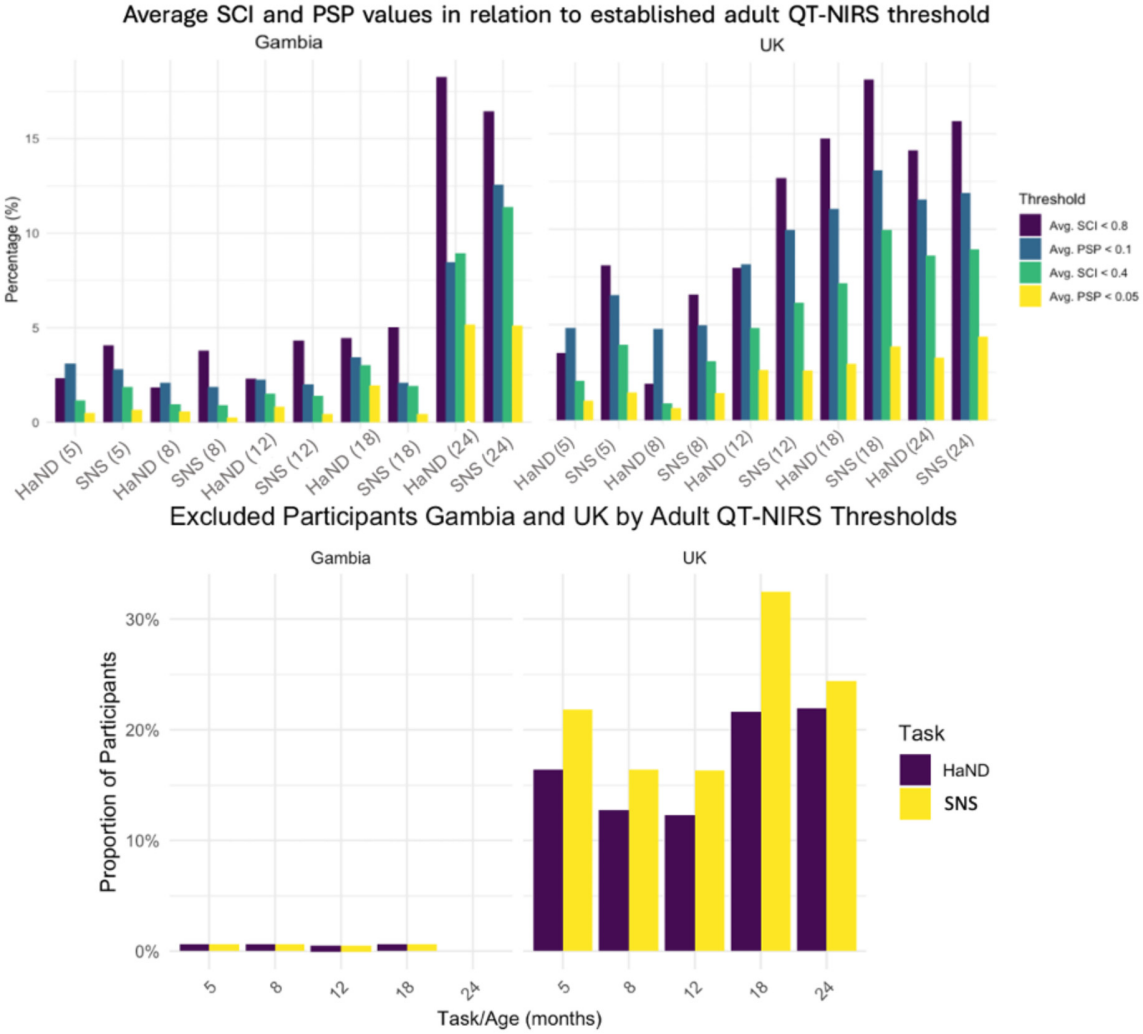
Data characteristics in relation to adult QT-NIRS thresholds. Top: bar charts showing the proportion of low channel average SCI and average PSP measures in relation to the adult recommended parameters of 0.8 (SCI) and 0.1 (PSP), and half of these threshold values (0.4 and 0.05, respectively). Top-left: Gambia cohort. Top-right: UK cohort. Bottom: number of participants, by age and task, with less than 60% of acceptable channels exhibiting mean SCI and PSP values compared with adult threshold values of 0.8 and 0.1, respectively. Highest exclusion rate was ∼33% for UK infants at 18 mo during the SNS task.

The psp_threshold value was varied, ranging from 0.005 to 0.1, with increments of 0.005. The upper value of 0.1 is the recommended threshold for adult participants. Values that spanned the entire possible range lower than this—based on initial results from simpler MLM analyses—were used; this is supported by the considerable proportion of infant PSP values lower than the adult threshold apparent during postanalysis data examination in the UK cohort (see Fig. 2).

##### Percentage of motion (P)

For each participant/age/task combination, the cross-channel mean of the percentage of windows per channel containing motion as identified by hmrMotionArtifactByChannel and subsequently excluded from analyses (see “Pruning”) was labeled the “Percentage of Motion” (PoM), providing a measure of the prevalence of motion.

##### Age (P)

Age was included as a five-level predictor (5-, 8-, 12-, 18-, 24mo) to assess change with age.

##### Cohort (C)

Cohort was included as a two-level covariate (Gambia and UK).

##### Task (C)

Task was included as a two-level covariate (HaND and SNS) to account for the contribution to variance of the different paradigms.

##### Channels pruned due to signal extrema (C)

The number of CSE per participant, for each age and task, was used as a participant-level covariate and proxy measure of poor optode coupling.

#### 2.3.2 Models and analyses

##### Comparison of QT-NIRS and CV pruning

In line with objective (a), each fNIRS recording underwent channel pruning using:

1. CV, by pruning channels where the CV values for different wavelength signals differed by 20% or more (“CV”),
2. sci_threshold only at every parameter value, by setting psp_threshold to 0, (“SCI Only”), and
3. Every combination of sci_threshold and psp_threshold parameters listed in Sec. 2.3.1 (“Full QT-NIRS”).

Pruning method 1 provides a baseline for the channel pruning, using a method and pruning criteria that have previously been used for the HaND task.^14^ Pruning approach 2 permits comparison of two time-domain methods (methods 1 and 2) to assess the impact of incorporating temporally specific measures. Pruning approach 3 provides insight into the benefit of additionally using a PSP threshold and pruning using frequency characteristics of the signal.

For every age/task/cohort combination, each sci_threshold parameter choice (SCI) or combination of sci- and psp_threshold values (QT-NIRS) was given two separate rankings according to their similarity to CV pruning, in terms of the mean number of channels retained across all participants and the total number of participants excluded. These two rankings were then combined to find the parameter (SCI) or parameters (QT-NIRS) producing the closest approximation to data retention provided by CV pruning.

For each of the 20 age (5 levels)/task (2 levels)/cohort (2 levels) combinations, the following model was then fitted to participant-level data:

TRC SNR∼Pruning Method+(1|ID), (1)

to compare the effect of pruning methods Eqs. (1)–(3) on signal quality whilst using a random intercept for each infant to account for inter-participant variability. To correct for multiple comparisons, p-values were Bonferroni-corrected.

##### Effect of SCI and PSP threshold choice on signal quality and retention

Models were designed to investigate the effect of sci_threshold, psp_threshold, age, and motion on TRC SNR and channels retained [Objectives (b) and (c)].

To examine potential combinations of theoretically viable predictors, covariates, and their interactions used for each outcome, a systematic approach to model building was used as a first step, combining predictors in various model formulas using combinatorial logic before assessing model fit. Model fit was assessed using the Akaike information criterion (AIC),^42^ a model selection criterion that balances goodness of fit with model complexity (Ref. 43, p. 824). Model variables were also included based on the outcomes of the subsidiary investigations described in Supplementary Material 3, which investigated the factors affecting motion incidence and average SCI and PSP measures at the channel level. These additional variables are included in the bottom line of Eq. (2).

Mindful of model convergence issues and overfitting, the priority when constructing models was to include predictors of interest, plus interaction terms, random slopes, and random intercepts that incorporated them. This resulted in the following model for both outcomes, TRC SNR and channels retained:

Outcome∼SCI*PSP+Age*SCI+Age*PSP+SCI*PoM+PSP*PoM+Age*PoM+Task+Cohort+CSE+(1|ID)+(1|SCI:Cohort)+(1|PSP:Cohort), (2)

where SCI = sci_threshold, PSP = psp_threshold, and the symbol “*” denotes an interaction term (itself denoted using “:”) plus all individual terms used in the interaction, as is consistent with the notation used in the lme4 package. Random intercept terms (bottom row) were included due to quantitative support based on subsidiary MLM investigations into average SCI and PSP measures.

Assessments of the model residuals showed that they were nonnormally distributed, so to calculate effects, a bootstrapping approach was used. Bootstrap datasets were generated by sampling rows from the original dataset with replacement, using a fixed random seed for reproducibility. The relevant model for each bootstrap sample was fitted using the lmer function from the lme4 package, and extracted fixed effect estimates. To mitigate potential biases caused by fitting models to data with different scales, variables $x_{i}$ were scaled and centerd

$$x_{i}^{\prime }=\frac{x_{i}-\overset{¯}{x}}{s_{x}},$$ (3)

where $x_{i}^{\prime }$ is the scaled value, and $\overset{¯}{x}$ and $s_{x}$ are the sample mean and standard deviation, respectively, of the variable x.

## 3 Results

**Fig. 3:**
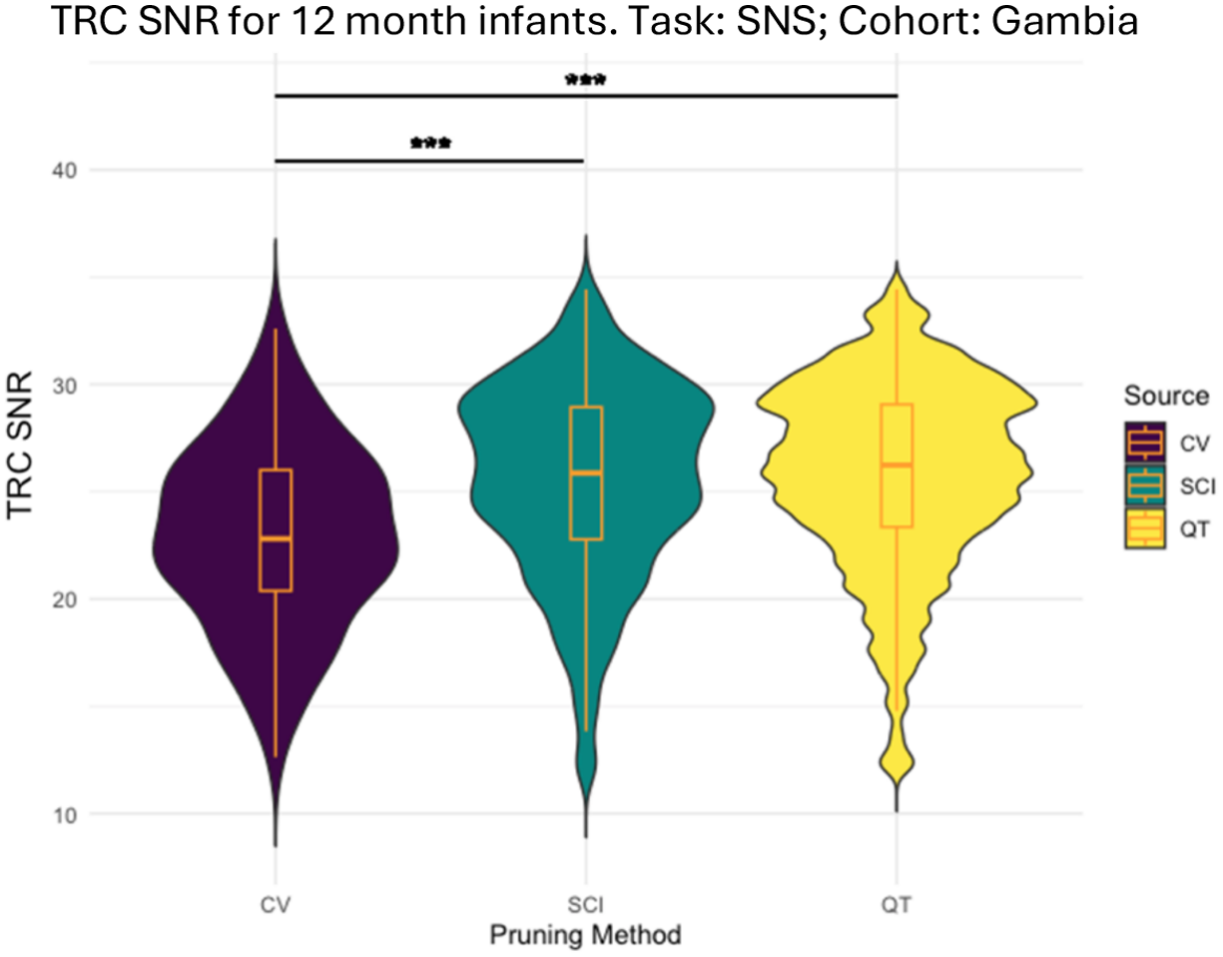
Example violin plot demonstrating typical differences between obtained mean TRC SNR values using CV pruning, QT-NIRS using SCI threshold only, and QT-NIRS utilizing both parameters for Gambian participants at 12mo during the SNS task.

CV pruning yielded an average TRC SNR of 23.7±1.66 across all 20 age/task/cohort combinations. Pruning with QT-NIRS using just the SCI threshold resulted in a mean increase of 2.13±0.644 in TRC SNR beyond that obtained using CV pruning. Similarly, using both SCI and PSP thresholding in combination resulted in a mean TRC SNR increase of 2.16±0.627 in comparison to CV pruning (see Fig. 3 and Supplementary Material 4 for the full comparison of TRC SNR values).

### 3.1 Comparison of QT-NIRS and CV Pruning

#### 3.1.1 Pruning using CV and QT-NIRS

The effect size and Bonferroni-corrected significance values of the three contrast conditions were calculated using Eq. (1); results are displayed in Table 1. For 19 of the 20 age/task/cohort combinations, significant (p<0.01) positive effects were found on the TRC SNR when controlling for data rejection when using QT-NIRS, using either the SCI only or full QT-NIRS approach. In 17 of 20 combinations, the differences were found to be statistically significant at the p<0.0001 threshold. Though the effect was positive for the single remaining combination of 20, it did not reach statistical significance.

**Table 1:** Condition contrasts between each of the three pruning methods. Methods are: CV, QT-NIRS using the sci_threshold parameter only, and QT-NIRS using both the sci_threshold and psp_threshold parameters. Significance values calculated during bootstrapping and reported after correction. Effect sizes rounded to 2d.p.

Age (months)	Task	Cohort	CV vs SCI only		CV vs. both parameters		SCI only vs. both parameters	
			Significance	Effect size	Significance	Effect size	Significance	Effect size
5	HaND	Gambia	<0.001	20.85	<0.001	20.83	1.0	0.05
8	HaND	Gambia	<0.001	12.71	<0.001	12.75	1.0	<0.01
12	HaND	Gambia	<0.001	19.32	<0.001	19.70	1.0	0.27
18	HaND	Gambia	<0.001	17.04	<0.001	17.10	1.0	0.04
24	HaND	Gambia	<0.001	12.36	<0.001	12.12	1.0	0.17
5	SNS	Gambia	<0.001	16.97	<0.001	16.93	1.0	0.02
8	SNS	Gambia	<0.001	14.69	<0.001	14.70	1.0	0
12	SNS	Gambia	<0.001	16.95	<0.001	17.65	1.0	0.49
18	SNS	Gambia	<0.001	15.10	<0.001	15.09	1.0	<0.01
24	SNS	Gambia	<0.001	13.38	<0.001	13.38	1.0	0
5	HaND	UK	<0.001	9.40	<0.001	9.40	1.0	<0.01
8	HaND	UK	<0.001	12.90	<0.001	12.90	1.0	<0.01
12	HaND	UK	<0.001	8.70	<0.001	8.56	1.0	0.10
18	HaND	UK	<0.001	10.92	<0.001	10.92	1.0	<0.01
24	HaND	UK	0.012	4.68	0.012	4.68	1.0	0
5	SNS	UK	<0.001	6.93	<0.001	7.80	1.0	0.62
8	SNS	UK	<0.001	7.18	<0.001	7.28	1.0	0.07
12	SNS	UK	0.924	3.23	0.288	3.87	1.0	0.45
18	SNS	UK	1.0	2.84	1.0	2.84	1.0	0
24	SNS	UK	1.0	0.76	1.0	0.76	1.0	<0.01

#### 3.1.2 Pruning using SCI only compared with both parameters

No significant statistical differences were found between TRC SNR values when pruning using SCI only and full QT-NIRS approaches. In every case, however, the mean TRC SNR across participants was higher when using both parameters than when using sci_threshold alone.

A representative comparison of the three different pruning methods is given in Fig. 3, with higher average TRC SNR values obtained using both QT-NIRS approaches when compared with CV pruning. A higher mean TRC SNR is obtained using both parameters when pruning with full QT-NIRS compared with using SCI only, but with a more erratic distribution of values.

### 3.2 Predictors of TRC SNR and Channels Retained

**Fig. 4:**
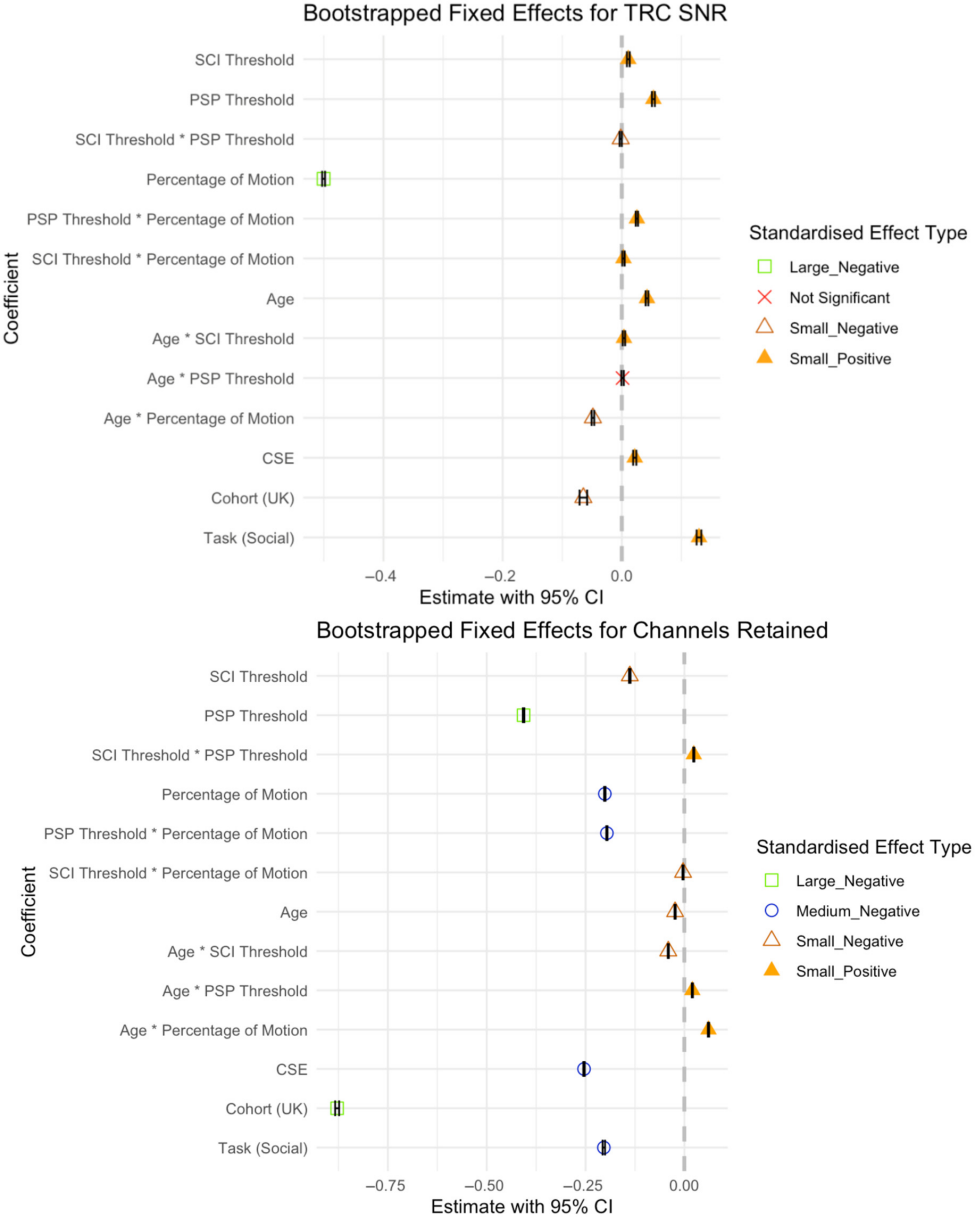
Forest plots showing the results for the effect of each fixed effect in Eq. (2) on TRC SNR and channels retained, obtained via bootstrapping scaled values. Top: Forest plot showing the type and relative effect size of each fixed effect on TRC SNR. Bottom: Forest plot showing the type and relative effect size of each fixed effect on Channels Retained.

The focus here is primarily on reporting results for the outcomes of interest and fixed effects with wider generalizability, but full results are included in Fig. 4. Effect sizes were classified as small, medium, or large if the absolute value of the estimate was <0.15, <0.35, or ≥0.35, respectively, using Cohen’s $f^{2}$ thresholds (Cohen, 2013^44^).

#### 3.2.1 Predictors for TRC SNR

Both SCI threshold (β=0.0107, 95% CI [0.0084,0.0131], SE=0.0012) and PSP threshold (β=0.0530, 95% CI [0.0505,0.0556], SE=0.0013) had small, positive effects representing an increase in signal quality for higher threshold values. The interaction effect between PSP threshold and SCI threshold had a small, negative effect (β=−0.0019, 95% CI [−0.0037,−0.0001], SE=0.0009).

**Fig. 5:**
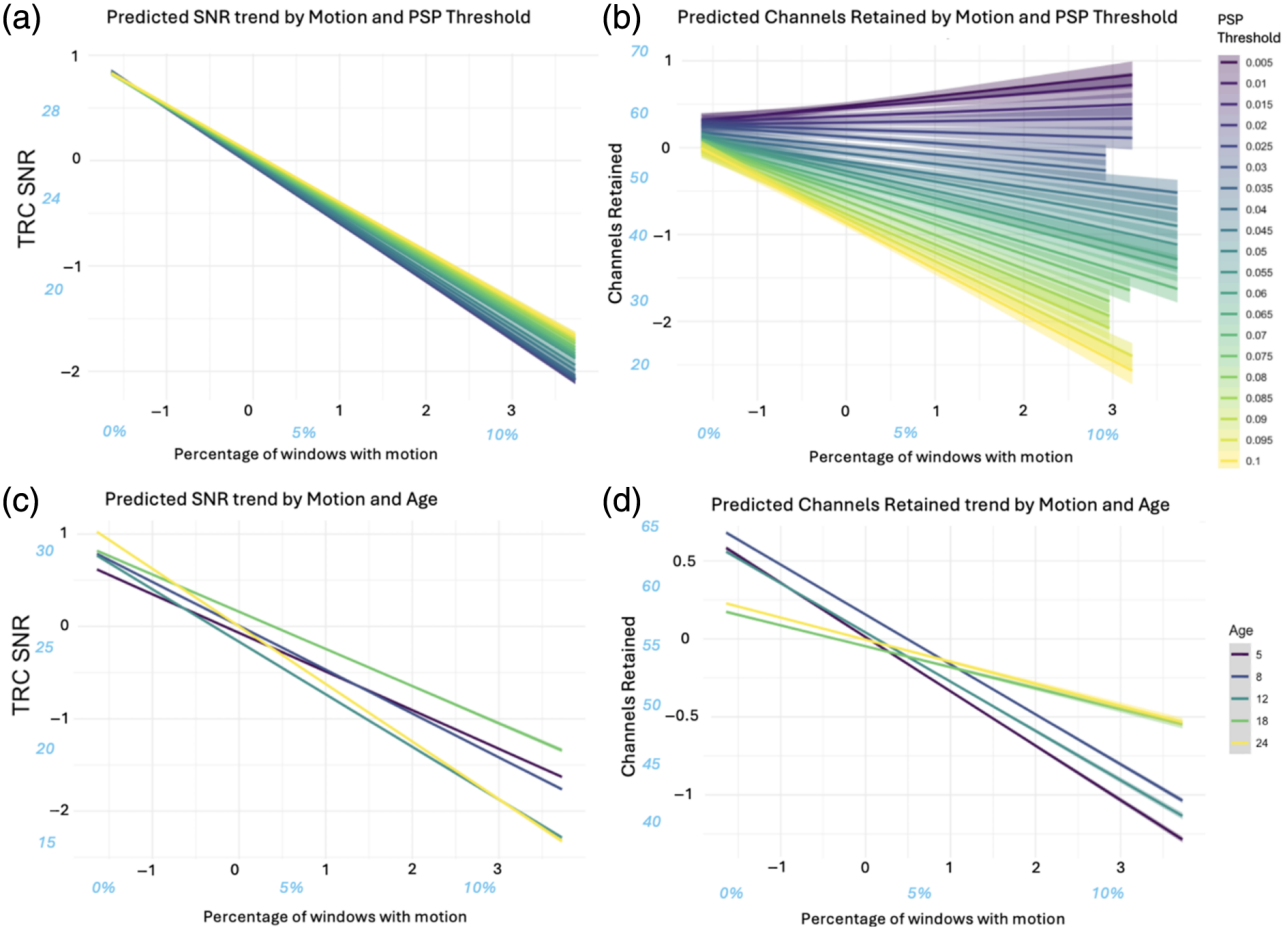
Relationship between motion, data quality, and retention. Blue, italicized axes values represent the approximate original values before scaling during analysis. (a) Predicted TRC SNR trend by percentage of motion, grouped by PSP threshold. (b) Predicted channels retained trend by percentage of motion, grouped by PSP threshold. (c) Predicted TRC SNR trend by percentage of motion, grouped by age. (d) Predicted channels retained trend by percentage of motion, grouped by age.

PoM had the largest impact on TRC SNR, with a large negative effect (β=−0.5004, 95% CI [−0.5028,−0.4979], SE=0.0012) corresponding to a decrease in signal quality in data with more frequent incidences of motion. This decrease in signal quality with motion was offset slightly as participants aged, driven mostly by a more moderate decrease in the 18mo data, illustrated by a small negative interaction effect between and PoM (β=−0.0485, 95% CI [−0.0506,−0.4062], SE=0.0011). Small interaction effects between PoM and both SCI threshold (β=0.0027, 95% CI [0.0008,0.0047], SE=0.0010) and PSP threshold (β=0.0253, 95% CI [0.0234,0.0272], SE=0.0010) exhibited trends that saw high threshold values mitigate the detrimental effect on TRC SNR; the slightly larger main (PSP) and interaction (PSP: PoM) effect in the case of PSP threshold led to greater mitigation of the TRC SNR decline due to PoM than in the case of SCI [see Fig. 5(a)].

**Fig. 6:**
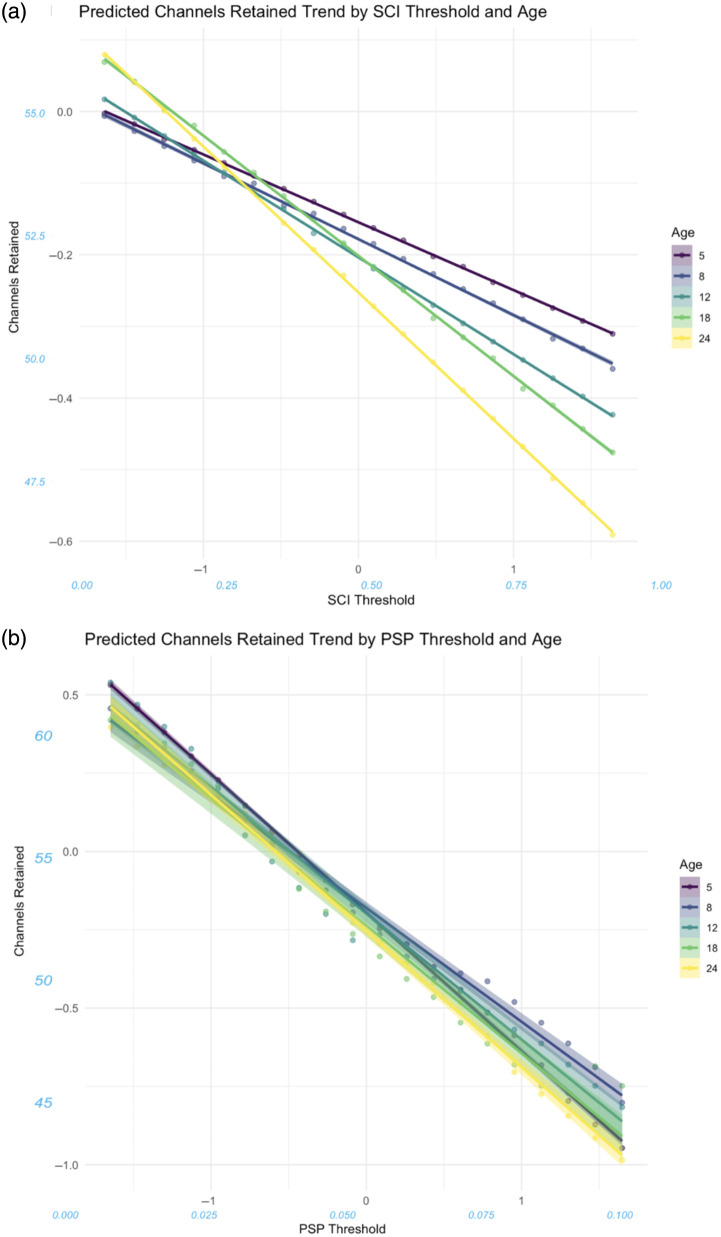
Effect of the interaction between QT-NIRS thresholds and age on channel retention. (a) The interaction between SCI threshold, showing the mitigating impact of high SCI values for younger participants. (b) The interaction between and PSP threshold, in which a pattern with age is harder to ascertain.

The small, negative effect of Cohort (β=−0.8783, 95% CI [−0.8832,−0.8733], SE=0.0025) is notable since this fixed effect had larger effects on other outcomes, including channels retained. The nonsignificant effect of PSP: age [Fig. 6(b)] is notable given the size of the dataset and the rarity of nonsignificant predictors throughout this work.

#### 3.2.2 Predictors for channels retained

**Fig. 7:**
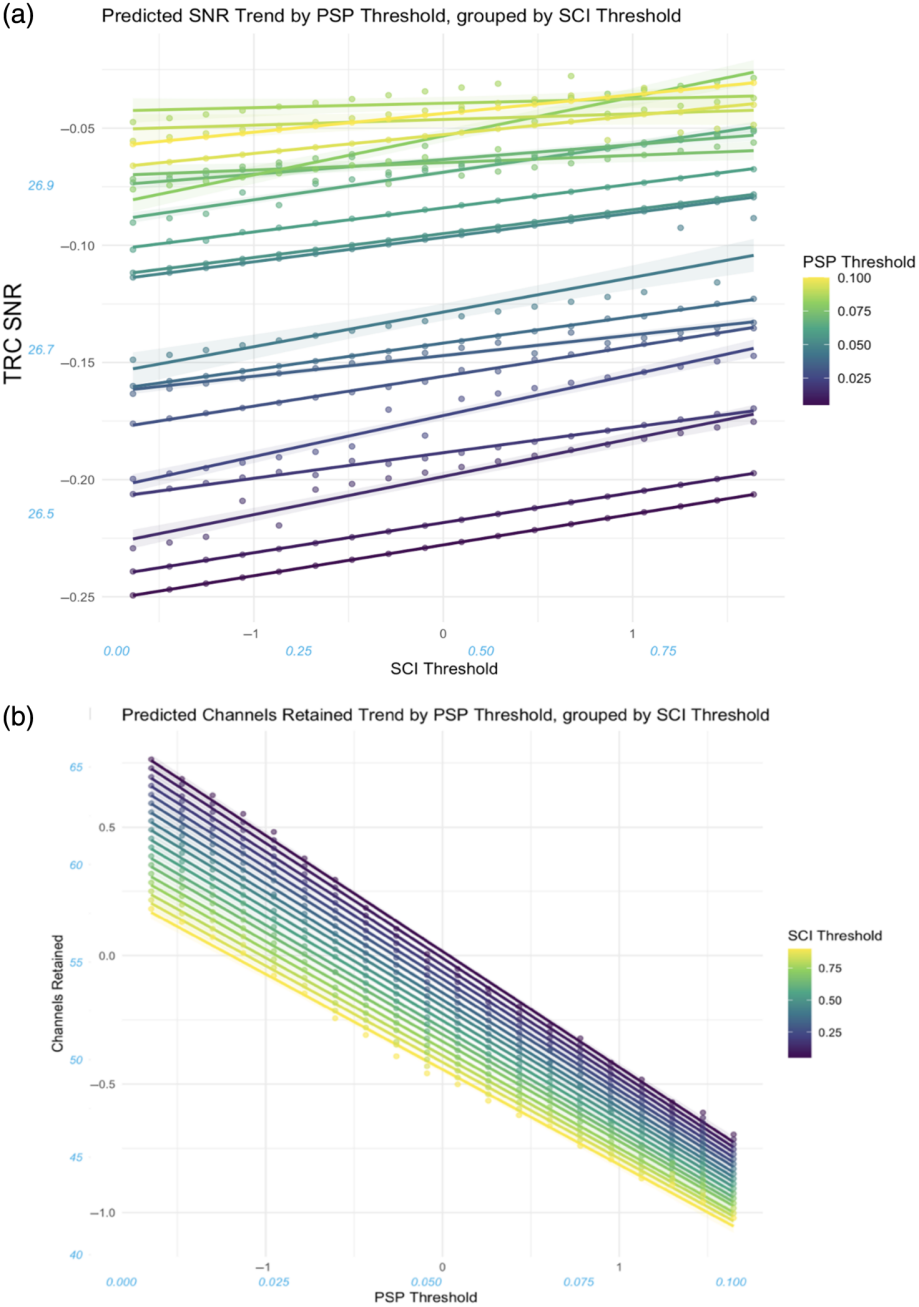
Effect of the interaction between SCI threshold and PSP threshold on signal quality and retention. (a) The interaction between thresholds acting on TRC SNR, showing an inconsistent, slight decrease in TRC SNR when compared with the overall trend for high SCI and PSP threshold values. (b) The interaction between thresholds acting on channel retained, showing a more consistent trend across threshold levels, and the dampening of the reduction in channels when both SCI and PSP threshold values are high.

Both SCI threshold (β=−0.1381, 95% CI [−0.1401,−0.1361], SE=0.0010) and PSP threshold (β=−0.4067, 95% CI [−0.4087,−0.4048], SE=0.0009) had small and large negative effects on channels retained, respectively (see Fig. 7). This indicates an increase in the number of channels pruned when using higher threshold values, particularly for the PSP threshold. The interaction effect between SCI threshold and PSP threshold was small and negative (β=−0.8783, 95% CI [−0.8832,−0.8733], SE=0.0025), with each threshold alleviating the negative effect of the other at high parameter values.

PoM had a significant, medium negative impact (β=−0.2013, 95% CI [−0.2030,−0.1995], SE=0.0009), with higher amounts of motion associated with decreased channel retention. At the two oldest ages (i.e., 18- and 24mo), greater proportions of motion in the data had a less drastic negative impact on the number of channels pruned [see Fig. 5(d)], which was captured by the small positive interaction effect Age: PoM (β=0.0610, 95% CI [0.0593,0.6264], SE=0.0009). As with TRC SNR, interaction effects of PoM with both SCI threshold (β=−0.0034, 95% CI [−0.0050,−0.0019], SE=0.0008) and PSP threshold (β=−0.1962, 95% CI [−0.1977,−0.1945], SE=0.0008) moderated this channel reduction for higher percentages of motion. Although both interactions were significant, PSP: PoM was of medium effect size and also acted on two (negative) medium main effects, leading to a near negation of the detrimental impact of motion on predicted channels retained for low PSP threshold values [see Fig. 5(b)].

Task (β=−0.2038, 95% CI [−0.2069,−0.2007], SE=0.0015) and cohort (β=−0.8783, 95% CI [−0.8832,−0.8733], SE=0.0025) had medium and large effects on channels retained, respectively, resulting in lower channel retention for the SNS task and UK cohort.

## 4 Discussion

Channel pruning in infant fNIRS research is a crucial step in data analysis, yet it is often performed with subjective parameters or adult-derived thresholds. Moreover, approaches to channel pruning, including QT-NIRS and CV pruning, in infant fNIRS data have often relied on adult-derived thresholds or single-domain measures, without systematic evaluation of their suitability for infant data. The work described here took a quantitative approach to directly compare pruning strategies and parameter settings for infant sparse array data. It was found that QT-NIRS, in its consideration of both time- and frequency-domain signal quality measures, achieved a better balance of data quality and retention than CV pruning. In addition, parameter values for QT-NIRS were investigated alongside contextual factors, with lower values than those used for adults likely being preferable. These findings supplement work in the wider literature aimed at improving infant NIR imaging data pipelines more broadly.^6^^,^^37^^,^^45^

### 4.1 QT-NIRS as a Channel Pruning Method

QT-NIRS produced infant fNIRS data of significantly greater SNR than pruning in all but one age/task/cohort-specific comparison (SNS task in UK infants at 24mo), whilst controlling for data retention. Higher signal quality remained statistically significant when channels were pruned using SCI only, suggesting that evaluating the signal using a method that accounts for a temporal data characteristic (correlation) is more effective than assessment using time-independent measures. It may also indicate that evaluation of the signal in subsampled windows provides a more accurate reflection of signal quality than holistic signal assessment, as was the case with CV pruning. This difference in signal quality, even without using PSP, is observed despite evidence suggesting that SCI may be biased by latent, undetected motion in the signal in at least some participants (see Sec. 4.3).

Using both SCI and PSP thresholds further improved signal quality, resulting in higher mean TRC SNR values in all cases. Although nonsignificant, the higher TRC SNR values reinforce the advantage of incorporating both frequency- and time-domain metrics and provide further justification for the use of QT-NIRS when channel pruning of infant fNIRS data.

### 4.2 Effects of QT-NIRS Parameter Choice

Higher SCI and PSP thresholds reduced data retention by pruning more channels but improved signal quality in TRCs. The effect of parameter changes was smaller than anticipated, particularly on signal quality. PSP threshold had more influence than SCI threshold on both outcomes, but particularly on channels retained, illustrating that caution is needed when altering this threshold to avoid unnecessary data loss.

A positive SCI: PSP interaction reduced the channel pruning rate relative to that expected when considering both thresholds in isolation [see Fig. 7(b)], suggesting that the potential cost to data retention from increasing one threshold value is mitigated when the other value is also high. This aligns with the use of complementary signal metrics in QT-NIRS, which must both be of sufficient quality to retain channels: each threshold will exclude channels that may be included by the other, with the overlap in excluded channels increasing with parameter values.

The slight negative SCI: PSP interaction effect on TRC SNR may be driven by the highest values for both parameters [Fig. 7(a)]. Higher thresholds increased the likelihood of pruning channels with neuronally evoked cortical hemodynamic responses and, consequently, high SNR; their removal is likely to reduce the mean TRC SNR more than would be expected for lower threshold values. Change in TRC SNR is also less uniform across SCI and PSP threshold values than for channels retained. Unlike channel retention, which reflects the whole array, TRC SNR is localized to select channels. Single channels will therefore likely contribute a much greater proportional value to the average TRC SNR, and the consequence of pruning these channels is likely to have a larger relative impact on the TRC SNR. In addition, pruning a channel consistently reduces channels retained, whereas its impact on TRC SNR depends on the pruned channel’s SNR, further contributing to the inconsistency in change.

### 4.3 Effects of Motion

Substantial negative effects of PoM on both signal quality and channel retention suggest that motion reduces signal quality in at least some channels even when artifacts are not considered in the pruning analysis process, as was the case in this work. Motion artifacts may cause optodes to move, dislodge, or uncouple completely, causing a poorer quality signal reflected in decreased TRC SNR. In turn, channels affected by motion may be pruned to a greater extent during QT-NIRS processing, leading to lower channels retained values.

Interaction effects show that PSP threshold had the greater impact on motion-affected data of the two thresholds, improving signal quality but reducing channel retention, especially at high threshold values. By contrast, a comparatively modest effect of SCI Threshold on signal quality and retention was found. Motion exhibited a significant adverse effect on average PSP, likely due to optode displacement severe enough to disrupt coupling, and a positive effect of motion incidences on average SCI, suggesting that SCI measures may be capturing correlation induced by latent, undetected motion artifacts in the data. Future work may examine the effect of different motion detection parameters, or alternative motion correction methods altogether, such as the Sobel filter,^46^ acceptance rate adaptive algorithm,^47^ global variance of temporal derivatives,^48^^,^^49^ or entropy-based methods.^50^

The potential (interaction) effect of high PSP threshold values on channel retention, especially for channels with a greater proportion of motion, warrants caution for users when looking to employ high PSP threshold values. This is particularly true given the relatively small beneficial impact on signal quality of increasing the PSP threshold, indicated by its main effect on TRC SNR. This is consistent with the argument for using lower values discussed in the Sec. 4.7 of the Sec. 4. Conversely, motion incidences have the largest negative impact on TRC SNR; low PSP thresholds may exacerbate this effect if motion is not appropriately addressed.

### 4.4 Effects of Age

Age had small effects on TRC SNR and channels retained, suggesting limited changes in signal quality and retention in infants between the ages of 5 and 24mo. Reflecting this, associated trends were less commonly observed with age than for other predictors; however, it was notable that channel loss was less severe when increasing the SCI threshold in younger participants (Fig. 6). In addition, although higher signal PoM is associated with reduced channel retention, older infants retained more channels than younger ones for data with high motion prevalence [Fig. 5(d)]. This may be due to younger infants manually touching or grabbing the cap more, causing more severe artifacts that permanently displace optodes and affect subsequent coupling, an assertion supported by posthoc data examination, which suggested a decrease in motion severity with age (see Fig. S7 in the Supplementary Material)

Interactions with age for TRC SNR showed no meaningful patterns. This may reflect a lack of consistent changes within the age range sampled here. It may also reflect the multifaceted mix of concepts that “age” represents: the interplay of the physiological and behavioral changes with age may be too complex for a simple fixed effect to capture. Interactions between age and other model terms (e.g., CSE and CL) caused convergence issues and were omitted. Future research may prioritize investigating age-related change and associated factors affecting signal quality—such as hair characteristics,^23^ hair style,^31^ or hair type changes with age.^51^

### 4.5 Other Predictors

#### 4.5.1 Cohort

Cohort had substantial effects on signal quality and retention, likely due to factors such as testing environment, tester experience, parent and infant behaviors, and sample size. Because the cohort was not a primary predictor, its main effect was not investigated in depth, and interaction terms were not included. Nevertheless, data from the Gambian cohort—despite skin and hair characteristics which have been found to pose challenges for fNIRS signal quality^23^—exhibited better signal quality and retention, to the extent that data exclusion was far lower for Gambian infants in general and almost nonexistent at the infant level (see Fig. 2). There may be several reasons for this. First, UK infants generally had finer hair, which was longer at later assessment ages, possibly causing cap slippage and poorer subsequent signal quality, as these values appear to decrease with age. Lower motion incidence in Gambian infants may also play a role, possibly due to a relative lack of familiarity with digital screens in daily life leading to an increased focus on a novel, unfamiliar object. Although physical characteristics of participants undoubtedly affect the fNIRS signal, cohort differences in this study emphasize the need to consider other factors that affect signal quality during data collection.

#### 4.5.2 Weak or saturated channel signals

A small and significant negative effect of CSE on channel retention was anticipated, considering that it is itself a measure of channel removal. CSE was also significantly negatively associated with data quality; however, suggesting poor coupling in channels with extremely low-quality signals could be affected by—or causing—signal quality issues elsewhere in the array. It may be of interest to investigate whether signal quality was worse in channels located closely to the CSE channels in future work as has been the focus of prior work into motion artifact detection.^50^

### 4.6 Strengths and Limitations

A key strength of this work is the dataset used. Data encompassed five testing time points across the age range 5- to 24mo, allowing assessment of QT-NIRS and its key parameters for infants whilst accounting for age-related structural changes in skull thickness, cardiac signal properties, and surface vasculature.^52^ Data from two sites were also assessed, one of which was a rural setting in a sub-Saharan African country, addressing a common bias that often exists in infant neuroimaging studies when participant recruitment is limited to predominantly white infants from high-income contexts.^53^ As such, the findings are likely to be more robust and generalizable than those derived from smaller or single-site samples. The inclusion of longitudinal data allows for the assessment of within- and between-participant changes over time, providing richer insights into the effect of processing methods than would be possible from cross-sectional analyses alone.

Another strength is the MLM approach, which accounted for the hierarchical variance structure of longitudinal data grouped by task and cohort, reducing bias and enabling random intercepts and slopes during regression analysis to capture individual differences.^39^ This approach also handled missing data introduced through prior channel removal (CSE channels), missed visits, or incomplete testing, which would not have been possible with many other common analysis procedures.^54^ Domain knowledge and data-driven insight were combined to prioritize model predictors and validate model assumptions, and additionally fitted and assessed subsidiary MLMs to ensure the final model [Eq. (2)] was as comprehensive as possible.

QT-NIRS was compared with CV pruning, using a maximum wavelength CV difference of 0.2, as previously applied with data from this study.^14^ Significant differences in signal quality between QT-NIRS and CV pruning were found in all but one age/task/cohort combination. However, another common CV pruning approach uses a single-channel threshold instead, pruning both channel wavelength signals at least one of them exceeds it.^12^^,^^32^^,^^55^ Future work could investigate whether the significant differences found here persist when using this alternative thresholding method.

The focus of this work was on optimizing thresholds of the two QT-NIRS parameters, which are most pivotal to performance, for which only reported recommended values for adult participants were found in the literature. Future work may focus on other parameters, such as changing the quality threshold (q_threshold), balancing channel quality discernment with the risk of losing nuance in signal characteristics. Future studies could also investigate the impact of altering window size or overlap: the former will likely balance measurement accuracy within windows against overall temporal sensitivity; the latter may provide more window temporal sensitivity at the expense of computational efficiency. Given the dominance of PSP threshold change on outcomes reported here, it may also be of interest to explore pruning using only the PSP threshold.^56^

This work must also be placed in the context of the increasing impact of machine learning on infant NIR imaging data processing, with future work in the field likely to assess the efficacy and generalizability of such methods. Deep learning approaches are frequently being added to the literature: a machine learning-based detector has been developed to identify “bad” channels to be pruned, for example.^56^ This approach was shown to be more adaptive, interpretable, and effective across diverse noise types than QT-NIRS and other methods, so future work may seek to assess the efficacy of this independently. In mitigation, care should be taken to ensure limitations common to many deep learning approaches (e.g., overfitting) are addressed through appropriate validation strategies, including the use of independent test sets and cross-validation.

Model design was limited by convergence issues, particularly when including random slopes and intercepts. The most likely and important sources of variation were prioritized; however, it was not possible to fully include all relevant interaction terms or random effects. In addition, linear terms were used in the models to simplify interpretability. Future work may incorporate nonlinear terms as an alternative to the bootstrapping approach for dealing with nonnormal residuals. Other alternative approaches could include sensitivity analysis of the influential points and outliers, variance modeling for specific predictors, and utilization of robust standard errors.

Although age is likely to reflect more than behavior and motion, the longitudinal study design may also result in participant familiarity with the testing procedures and the paradigms, in turn possibly affecting behavior, attention, stress levels, engagement, and—consequently—the recorded data. Thus, caution should be taken when examining the effects of the age model term and its interactions, recognizing this as a potential confounding factor in this work.

Further evaluation of QT-NIRS as a channel pruning method for infant fNIRS data is still necessary to address the limitations of this work, extend it to high-density systems, and compare it to alternative approaches including those incorporating machine learning.^56^

### 4.7 Recommendations

Based on this work, guidelines for channel pruning of infant fNIRS data are given in Table 2.

**Table 2:** Recommendations for channel pruning of infant NIR imaging data based on the outcomes of this work.

Recommendation	Rationale
Use QT-NIRS for channel pruning as it is preferable to pruning using CV (when using a minimum threshold difference between wavelengths)	QT-NIRS achieved higher TRC SNR than CV pruning in nearly all age/task/cohort comparisons, even when using SCI alone, indicating that infant channel quality is better captured by methods combining temporal and frequency-domain information and/or a windowed approach
Conduct channel pruning on motion-free data	Pruning after removing motion artifacts ensures channel decisions reflect true optical signal quality
Prioritize tuning the QT-NIRS PSP threshold over the SCI threshold	Changing the PSP threshold showed a larger influence on both data quality and retention than changing the SCI threshold, especially in the presence of motion
Use lower QT-NIRS pruning thresholds for infant data than adult recommendations (minima of psp_threshold ≈0.04 to 0.05 and sci_threshold ≈0.6), especially for infants with fine/slippery hair	(i) A large proportion of data used in this work exhibited SCI and PSP values lower than the adult thresholds of 0.8 and 0.1, respectively (Fig. 2)
	(ii) The risk of removing data with higher thresholds is likely greater than the potential gain in improved signal quality
	(iii) There is a plateau in both average TRC SNR and channels retained values when using lower threshold values than the advised minima (see Fig. S6 in the Supplementary Material)

**Fig. 8:**
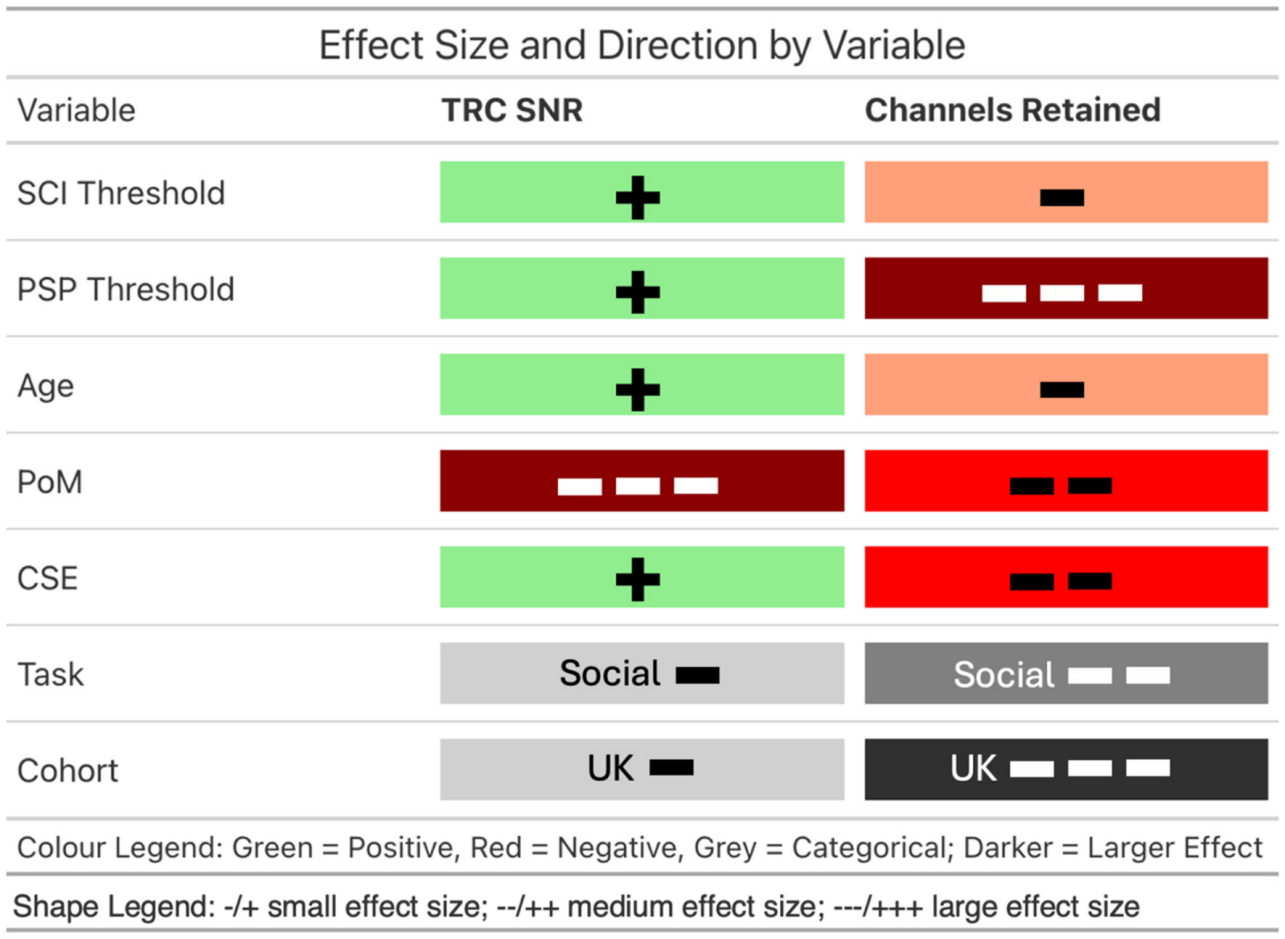
Guide to the effects of the parameters and data characteristics on data quality and retention. First five rows represent positive (green) or negative (red) associations with increasing numerical parameters (SCI threshold and PSP threshold) or data characteristics (age, PoM, and CSE). All positive effects are small in size. Bottom two rows represent categorical variables, with grayscale shading indicative of the impact on the outcome that changing the categorical variable may have.

A pictorial guide to the effect of the most common predictors on data quality and retention is also included in Fig. 8.

It is still strongly recommended that fNIRS users take the appropriate time to understand the dataset being analyzed beacuse no approach can possibly be universal. To aid final parameter selection, a tool that other users may find useful to guide parameter selection was developed. Alongside code used during processing and analysis reported in this study can be found at: https://github.com/sam-beaton/pruningComparisons/. The tool is designed to examine the effects of parameter threshold changes within a group (e.g., age), by establishing a trend capturing the trade-off between data quality and retention, then assessing that specific SCI and PSP threshold parameter combinations perform best in relation to this trend.

## 5 Conclusion

Recent advances in fNIRS channel pruning approaches show promise for improving the accuracy of preprocessing by evaluating both the time and frequency domains. The work described here compared QT-NIRS with an established pruning method utilizing CV across five infant ages, two paradigms, and two sites. It was found that QT-NIRS provides data with greater signal quality when controlling for data retention. The consequences of different parameter choices for QT-NIRS were also demonstrated, highlighting the importance of the PSP threshold, plus the influence of motion. Evidence-based recommendations for QT-NIRS pruning, and parameter choice for infant data with different characteristics, are provided.

## Supplementary Material

10.1117/1.NPh.13.1.015011.s01

## Data Availability

All data used in the analyses presented can be made available following relevant approvals. The code used to conduct the analyses and generate the figures presented in this paper is available at https://github.com/globalfnirs/pruningComparisons. Code relies on the following open-source R libraries: arules,^57^ broom,^58^ car,^59^ data,^60^ doParallel,^61^ dplyr,^62^ effectsize,^63^ effects,^59^ e1071,^64^ foreach,^65^ ggplot2,^66^ ggpubr,^67^ gridExtra,^68^ lme4,^41^ lmerTest,^69^ MASS,^70^ matrix,^71^ MuMIn,^72^ Scales,^73^ tidyr,^74^ and viridis.^75^ The data used to support this study are stored in the Brain Imaging for Global Health Data Repository. The conditions of ethics approval do not allow public archiving of pseudo-anonymized study data. The data cannot be fully anonymized due to the nature of combined sources of information, such as neuroimaging, sociodemographic, geographic, and health measures, making it possible to attribute data to specific individuals. Hence, the release of data would not be compliant with GDPR guidelines unless additional participant consent forms are completed.^26^ Access to any data collected during or generated by the BRIGHT project is fully audited and, to ensure data security, is overseen by the data management team in the UK and The Gambia. Relevant data sharing procedures were created in consultation with stakeholders and external consultation (Begum Ali & Holman, 2023^76^). To access the data, interested readers should use the contact page of the BRIGHT website. Access will be granted to named individuals following ethical procedures governing the reuse of sensitive data. Specifically, requestors must preregister their proposal and clearly explain the planned analysis to ensure that the purpose and nature of the research are consistent with that to which participating families originally consented. In addition, requestors must complete and sign a data sharing agreement to ensure data is stored securely. Approved projects would need to adhere to the BRIGHT project’s policies on ethics, data sharing, authorship, and publication.
